# Supplementary material for: MR1-Restricted MAIT Cells From The Human Lung Mucosal Surface Have Distinct Phenotypic, Functional, and Transcriptomic Features That Are Preserved in HIV Infection
Source: Front Immunol. 2021 Apr 9;12:631410. doi: 10.3389/fimmu.2021.631410 (PMC8062704; doi:10.3389/fimmu.2021.631410)
Supplement: Supplementary file 1 [file DataSheet_1.pdf]

Supplementary Material

Supplementary Table 1: MAIT tissue repair genes also upregulated by bronchoalveolar MAIT cells in comparison to peripheral blood MAIT cells.

Gene	Log_2Fold Change	Adjusted *P*
IL1B	5.4659	0.0119
CXCL10	5.3120	0.0871
JAG2	4.6732	0.0926
PMP22	4.4515	0.0644
CXCL2	4.4232	0.0616
TNFRSF21	4.0764	0.0918
CSF2	4.0119	0.1972
HBEGF	3.9078	0.1008
INHBA	3.8365	0.2714
FLG	3.5022	0.2018
APOE	3.3867	0.2262
WNT10A	3.1996	0.2962
ADM	2.7396	0.5015
ZBTB7C	2.3640	0.4231
ENG	2.0479	0.1762
LGALS3	2.0072	0.0746
ADAMTS2	1.7944	0.2769
SYK	1.7857	0.1743
CXCL12	1.5037	0.6055
IGF1	1.2513	0.1127
CSF1R	1.2232	0.6028
PDGFA	1.0010	0.6487
THBS1	0.9028	0.5151
FGFR2	0.8919	0.2309
CSF1	0.7786	0.5034
CCL3	0.7190	0.6362
BMP7	0.6525	0.3417
DISP1	0.5697	0.7446
EREG	0.5116	0.5041
FLG2	0.4911	0.6169
LEP	0.4810	0.7144
EPGN	0.4482	0.5569
WNT7B	0.3903	0.6004
APP	0.3797	0.8234
ANGPT2	0.2230	0.7109
CRISPLD2	0.1924	0.8829

HIF1A	0.1474	0.9027
IFT172	0.0849	0.9665
VEGFB	0.0816	0.9475

Supplementary Table 2: Genes differentially expressed by MR1 tetramer-negative TRAV1-2+CD161+CD8+ T cells cells during latent TB infection and similarly differentially expressed by bronchoalveolar MAIT cells as compared to peripheral blood MAIT cells.

**Downregulated genes**			**Upregulated genes**		
Gene	Log_2Fold Change	Adjusted *P*	Gene	Log_2Fold Change	Adjusted *P*
SHQ1	-2.6701	0.0284	*CD4*	4.2899	0.0694
UCHL5	-2.1101	0.0491	*MYLK4*	3.0350	0.0921
DCP1B	-1.7564	0.1866	*TNFAIP2*	2.7483	0.2181
KIAA0922	-1.3915	0.2070	*CST3*	2.5643	0.2736
GOT2	-1.3571	0.2150	*HLA-DRB6*	2.5482	0.0698
CLPTM1	-1.3420	0.1664	*PTAFR*	2.5395	0.0009
REXO4	-1.3369	0.2481	*SYK*	1.7857	0.1743
DUS2L	-1.2888	0.4092	*EMR2*	1.7575	0.1492
DYM	-1.2671	0.2520	*DYDC1*	1.6938	0.1745
UAP1	-1.2588	0.2778	*LOC148709*	1.6510	0.2260
ADK	-1.2569	0.2680	*UCKL1-AS1*	1.2333	0.1327
SF3B4	-1.2379	0.2404	*FFAR2*	1.2208	0.1693
G6PD	-1.1753	0.2812	*TMEM170B*	1.1443	0.1714
SUCLG2	-1.1519	0.3105	*LOC284379*	1.1325	0.1008
TBC1D14	-1.0872	0.3873	*POU5F1*	1.1270	0.1433
EHMT1	-1.0569	0.2885	*PARD6G*	1.1220	0.0832
C17orf62	-1.0178	0.3102	*C4orf26*	1.1031	0.1397
DDB1	-1.0110	0.3546	*INMT*	1.0740	0.1059
PSMC4	-0.9982	0.3227	*NLRP12*	1.0387	0.1513
MDH2	-0.9210	0.3924	*ITGA2*	1.0100	0.2177
FNTA	-0.8901	0.4044	*SLC16A12*	0.9879	0.3541
NFYB	-0.8863	0.3046	*LOC100506385*	0.9696	0.2255
P4HTM	-0.8772	0.4338	*CNNM1*	0.9499	0.2671
SAE1	-0.8015	0.4571	*BHMT2*	0.9432	0.1588
RHBDD2	-0.7844	0.4511	*MBOAT2*	0.9405	0.2610
PSMC2	-0.7532	0.4045	*NDST3*	0.8965	0.2561
DENND2D	-0.6513	0.5297	*C9orf66*	0.8714	0.2814
KCNA3	-0.6052	0.5338	*IL17RD*	0.8516	0.2422
CASP8	-0.5869	0.2509	*GSTTP2*	0.8512	0.3037

AP3S2	-0.5843	0.4798	*FBLIM1*	0.8419	0.1897
KDELR2	-0.5626	0.5786	*LOC100292680*	0.8375	0.5657
YTHDF2	-0.5545	0.5784	*FOXP4*	0.8366	0.2492
ARCN1	-0.4939	0.4761	*ADCY1*	0.8257	0.3401
ATF2	-0.4807	0.6711	*PART1*	0.8200	0.2501
HADHB	-0.4558	0.6642	*EMX2OS*	0.8016	0.2076
SSR1	-0.3165	0.4241	*TMEM17*	0.7826	0.3050
CCR1	-0.3157	0.8277	*IAPP*	0.7728	0.2636
ACTR3	-0.1953	0.8609	*C21orf62*	0.7703	0.1990
ACAD11	-0.1938	0.8798	*LOC100129269*	0.7659	0.2206
CNDP2	-0.1604	0.8786	*S1PR3*	0.7407	0.4457
PTPN22	-0.1474	0.8947	*C14orf105*	0.7352	0.3690
-	-	-	*RAB3B*	0.7261	0.2027
-	-	-	*PTK6*	0.7249	0.2904
-	-	-	*MSRB3*	0.7112	0.2961
-	-	-	*C1orf140*	0.7106	0.3481
-	-	-	*LOC100128338*	0.7095	0.2399
-	-	-	*FRRS1*	0.7081	0.3074
-	-	-	*ST6GAL2*	0.7039	0.3916
-	-	-	*FCAR*	0.7026	0.2764
-	-	-	*PSME4*	0.6888	0.4503
-	-	-	*AGMO*	0.6763	0.4114
-	-	-	*OLFML2A*	0.6762	0.2584
-	-	-	*CEACAM8*	0.6610	0.4789
-	-	-	*KREMEN1*	0.6568	0.2485
-	-	-	*LOC100287792*	0.6423	0.2394
-	-	-	*SYNPO2*	0.6343	0.3566
-	-	-	*LAMC2*	0.6269	0.5295
-	-	-	*TLCD2*	0.5985	0.2482
-	-	-	*CABP4*	0.5924	0.2043
-	-	-	*IRGQ*	0.5852	0.1588
-	-	-	*TTL*	0.5747	0.6595
-	-	-	*VSTM4*	0.5738	0.3236
-	-	-	*TRIM58*	0.5723	0.2671
-	-	-	*PDE6A*	0.5612	0.3774
-	-	-	*LOC100128682*	0.5426	0.2764
-	-	-	*LOC729603*	0.5388	0.3410
-	-	-	*CACNG8*	0.5231	0.2724
-	-	-	*EMP2*	0.5182	0.3263
-	-	-	*CEACAM22P*	0.4735	0.3803
-	-	-	*MYLK3*	0.4354	0.3615
-	-	-	*FLJ43879*	0.4261	0.4326
-	-	-	*LOC284950*	0.4236	0.4711
-	-	-	*CHST6*	0.3997	0.4104
-	-	-	*SLC36A2*	0.3795	0.5738
-	-	-	*POM121L10P*	0.3724	0.4466

-	-	-	*LOC100128288*	0.3585	0.5745
-	-	-	*KLB*	0.3401	0.5633
-	-	-	*NWD1*	0.3397	0.5703
-	-	-	*SLC15A2*	0.2983	0.6820
-	-	-	*FKBP9*	0.2884	0.6537
-	-	-	*ARGFX*	0.2784	0.4804
-	-	-	*CPA4*	0.2259	0.7484
-	-	-	*NAPSB*	0.2116	0.9262
-	-	-	*TSIX*	0.2041	0.7849
-	-	-	*ARSD*	0.1710	0.8481
-	-	-	*C3orf62*	0.1082	0.8512
-	-	-	*LOC400548*	0.0885	0.9055
-	-	-	*FHDC1*	0.0711	0.9386
-	-	-	*LOC286437*	0.0688	0.9345
-	-	-	*AFF3*	0.0464	0.9434
-	-	-	*S100PBP*	0.0087	0.9931

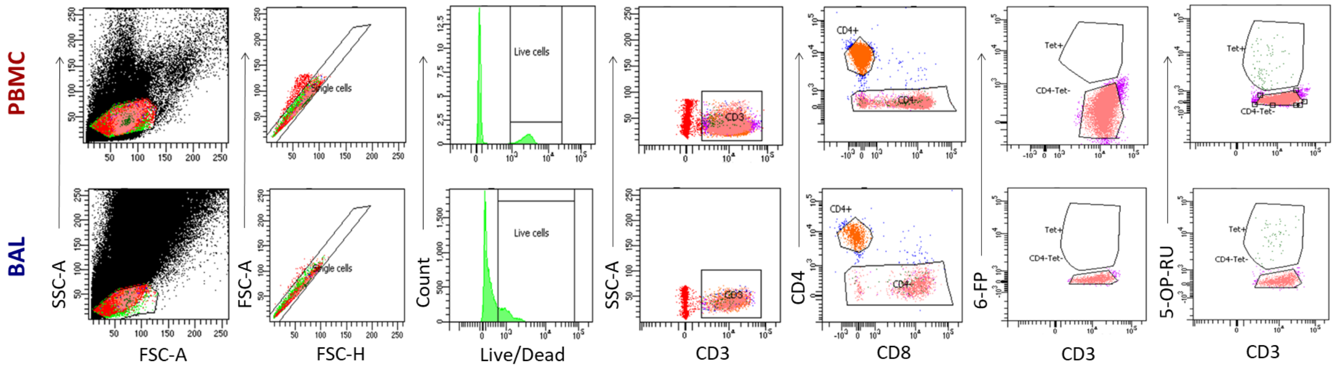

Supplementary Figure 1: Gating strategy used to sort MR1 tetramer-positive MAIT cells for RNA-sequencing. Cells were sorted from the peripheral blood and bronchoalveolar compartments using the MR1 6-FP tetramer to define the MR1 5-OP-RU tetramer gate.

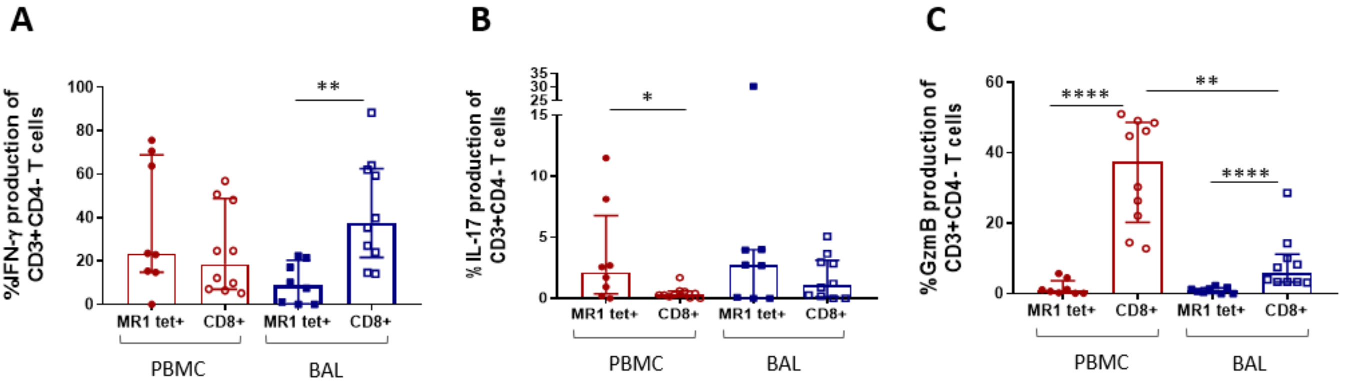

Supplementary Figure 2: Pro-inflammatory cytokine and cytolytic molecule production of MR1 tetramer-positive MAIT cells (n = 8) in the peripheral blood (red) and bronchoalveolar lavage (BAL) fluid (blue) in contrast to matched conventional CD8+ T cells (n = 10) from healthy participants showing (**A**) inducible IFN-γ, (**B**) inducible IL-17 and (**C**) constitutive granzyme B production. Bars represent medians and error bars represent interquartile ranges. Statistical difference was determined using the Mann-Whitney *U* test.

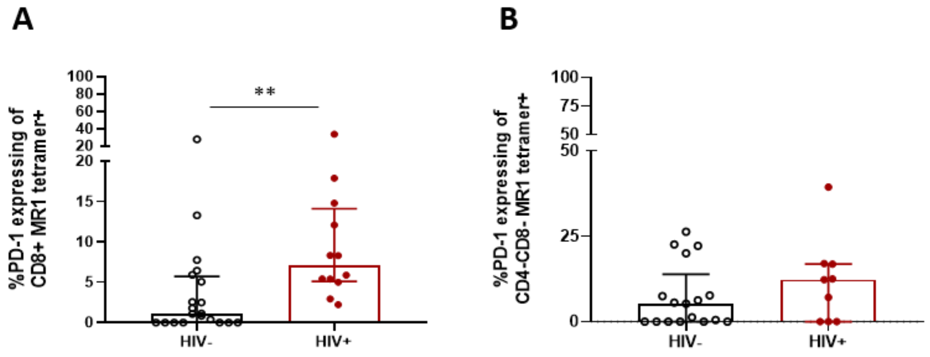

Supplementary Figure 3: Frequency of PD-1 expressing (**A**) CD8+ MR1 tetramer-positive MAIT cells and (**B**) CD4-CD8- MR1 tetramer-positive MAIT cells from the peripheral blood of HIV-negative and HIV-positive participants. Bars represent medians and error bars represent interquartile ranges. Statistical difference was determined using the Mann-Whitney *U* test.

A

	Cluster	PBMC	BAL	HIV-	HIV+
	BAL_1	24.56%	75.44%	35.09%	64.91%
	PBMC_1	86.54%	13.46%	34.62%	65.38%
	PBMC_2	64.58%	35.42%	45.83%	54.17%
	BAL_2	30.30%	69.70%	60.61%	39.39%

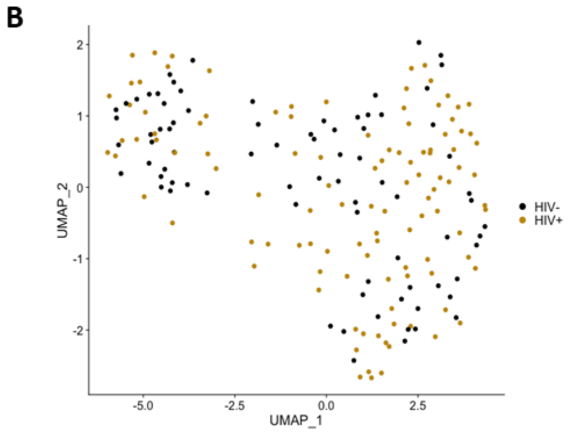

Supplementary Figure 4: (**A**) Summary of characteristics of MR1 tetramer-positive MAIT cell transcriptomic clusters. (**B**) UMAP plot showing assignment of HIV status to MAIT cell transcriptomic subsets.

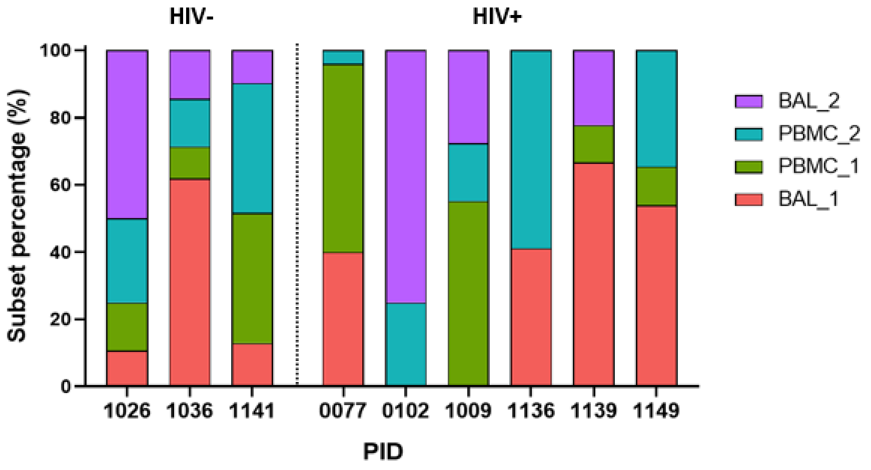

Supplementary Figure 5: Percentage distribution of MAIT cell transcriptomic subsets by each participant identifier (PID) from both the peripheral blood and bronchoalveolar lavage fluid.

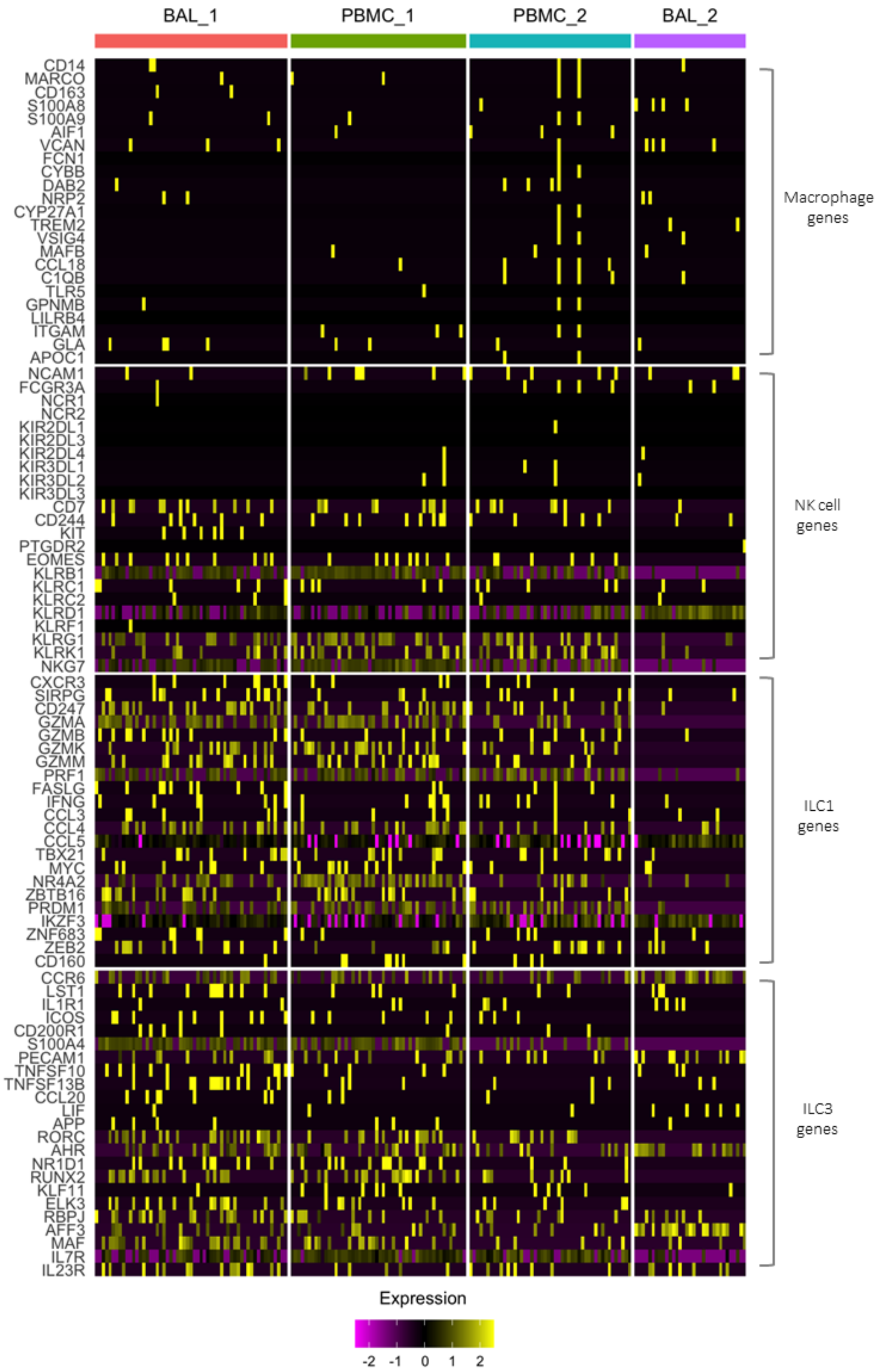

Supplementary Figure 6: Heatmap showing the expression of canonical macrophage, natural killer (NK) cell, and group 1 and group 3 innate lymphoid cell (ILC) genes across the four transcriptional MAIT cell subsets.

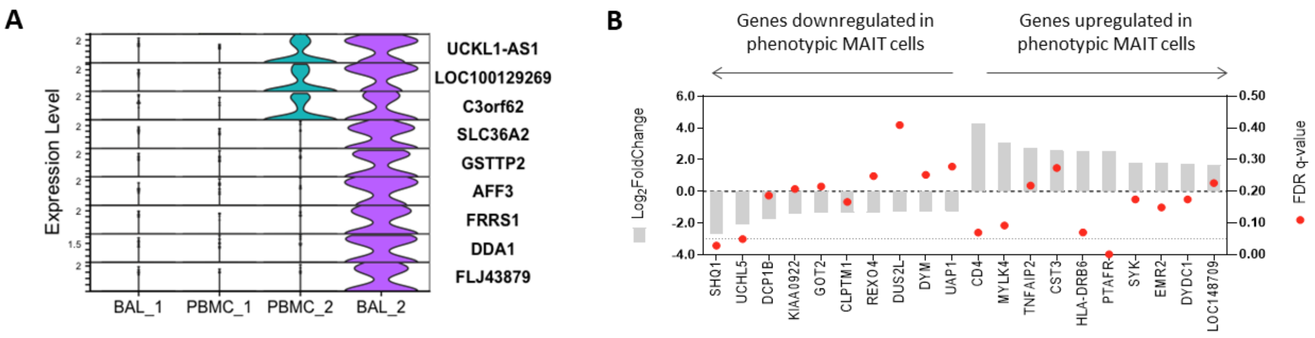

Supplementary Figure 7: A) Violin plot showing the expression of TB-specific TRAV1-2+CD161++CD8+ T cell genes by MAIT cell transcriptional subsets. **B**) Bulk RNA-sequence analysis showing the enrichment of TRAV1-2+CD161++CD8+ T cell genes in bronchoalveolar versus peripheral blood MAIT cells of HIV-negative individuals.
